# Functional Expression of the Human Glucose Transporters GLUT2 and GLUT3 in Yeast Offers Novel Screening Systems for GLUT-Targeting Drugs

**DOI:** 10.3389/fmolb.2020.598419

**Published:** 2021-02-18

**Authors:** Sina Schmidl, Sebastian A. Tamayo Rojas, Cristina V. Iancu, Jun-Yong Choe, Mislav Oreb

**Affiliations:** ^1^ Institute of Molecular Biosciences, Faculty of Biological Sciences, Goethe University Frankfurt, Frankfurt am Main, Germany; ^2^ Department of Chemistry, East Carolina Diabetes and Obesity Institute, East Carolina University, Greenville, NC, United States; ^3^ Department of Biochemistry and Molecular Biology, The Chicago Medical School, Rosalind Franklin University of Medicine and Science, North Chicago, IL, United States

**Keywords:** GLUT2, GLUT3, Glucose transport inhibitor, drug screening system, hxt^0^ yeast strain

## Abstract

Human GLUT2 and GLUT3, members of the GLUT/SLC2 gene family, facilitate glucose transport in specific tissues. Their malfunction or misregulation is associated with serious diseases, including diabetes, metabolic syndrome, and cancer. Despite being promising drug targets, GLUTs have only a few specific inhibitors. To identify and characterize potential GLUT2 and GLUT3 ligands, we developed a whole-cell system based on a yeast strain deficient in hexose uptake, whose growth defect on glucose can be rescued by the functional expression of human transporters. The simplicity of handling yeast cells makes this platform convenient for screening potential GLUT2 and GLUT3 inhibitors in a growth-based manner, amenable to high-throughput approaches. Moreover, our expression system is less laborious for detailed kinetic characterization of inhibitors than alternative methods such as the preparation of proteoliposomes or uptake assays in *Xenopus* oocytes. We show that functional expression of GLUT2 in yeast requires the deletion of the extended extracellular loop connecting transmembrane domains TM1 and TM2, which appears to negatively affect the trafficking of the transporter in the heterologous expression system. Furthermore, single amino acid substitutions at specific positions of the transporter sequence appear to positively affect the functionality of both GLUT2 and GLUT3 in yeast. We show that these variants are sensitive to known inhibitors phloretin and quercetin, demonstrating the potential of our expression systems to significantly accelerate the discovery of compounds that modulate the hexose transport activity of GLUT2 and GLUT3.

## Introduction

Transport of hexoses across plasma membranes marks the first and rate-limiting step of energy metabolism in cells of all domains of life. In humans, 14 glucose transporter family members (GLUTs, SLC2 gene family), with differing tissue distributions, mediate the facilitative diffusion of sugar along a concentration gradient. Despite a high sequence similarity, GLUTs differ in substrate specificity and affinity (Mueckler and Thorens, 2013), matching the demands for complex, tissue-dependent hexose uptake. Abnormal expression, localization or function of GLUTs are related to the pathogenesis of several diseases including cancer (Barron et al., 2016), diabetes (Ohtsubo et al., 2005; Hajiaghaalipour et al., 2015), and other severe metabolic disorders (Santer et al., 1997; Brockmann, 2009), making these transporters important drug targets.

GLUT2 is the primary GLUT isoform found in the liver. It also mediates glucose transport in the kidney, intestine, pancreatic β-cells and the central nervous system (Fukumoto et al., 1988; Thorens, 2015). It exhibits a low affinity for glucose (*K*_*M*_ = ∼17 mM (Uldry et al., 2002)) and even lower for fructose, galactose, and mannose (*K*_*M*_ = ∼76, ∼92, and ∼125 mM, respectively (Mueckler and Thorens, 2013)). For glucosamine, however, GLUT2 shows a very high substrate affinity (*K*_*M*_ = ∼0.8 mM) (Uldry et al., 2002). Under normal physiological conditions, the glucose concentration in human blood is ∼5.5 mM (Jung et al., 2013); the glucose uptake by GLUT2 would be inefficient, indicating that it is not the primary function of this transporter. More likely, glucose sensing (Ohtsubo et al., 2005) and/or signaling (Guillemain et al., 2000) are the main functions of GLUT2, consistent with its expression in tissues with high glucose fluxes. In murine pancreatic β-cells, GLUT2 mediates glucose stimulated insulin secretion, thereby regulating blood glucose levels. The absence of this function impairs glucose homeostasis leading to diabetes (Ohtsubo et al., 2005). Furthermore, GLUT2 may also be involved in mediating transcriptional glucose signaling (Guillemain et al., 2000). Loss of GLUT2 function causes the Fanconi Bickel Syndrome (Santer et al., 1997), a rare autosomal disease with various symptoms like hepatomegaly, tubular nephropathy, glucose and galactose intolerance, fasting hypoglycemia, rickets, and retarded growth (Santer et al., 2002).

In contrast to GLUT2, GLUT3 exhibits a high affinity for glucose (*K*_*M*_ = 1.4 mM (Colville et al., 1993)). Other substrates of GLUT3 are mannose, galactose, and xylose (Simpson et al., 2008). GLUT3 shares a high sequence identity (66%) with GLUT1 (Deng et al., 2015), and together they are predominantly responsible for glucose uptake in the brain - GLUT1 in the blood-brain barrier and GLUT3 in neurons (Simpson et al., 2007). Accordant with its high affinity for glucose, GLUT3 plays a pivotal role in the glucose uptake of cell types with a high demand for energy, such as sperm, circulating white blood cells, and preimplantation embryos (Simpson et al., 2007). Consistently, as tumor tissues have an increased need for carbon sources to sustain uncontrolled proliferation, GLUT3 is upregulated in many cancers, including gliomas, lung, laryngeal and bladder tumors (Ancey et al., 2018; Barron et al., 2016). In general, high expression of GLUT3 (and GLUT1) is associated with severe pathogenesis and poor survival in most cancer tissues (Ancey et al., 2018). Recently, an association between diverse diseases and variations of copy numbers of the GLUT3 gene has been hypothesized (Ziegler et al., 2020). Hence, the discovery of activating or inhibiting drugs specific for GLUT2 or GLUT3 is highly desirable.

Both transporters GLUT2 and GLUT3 belong to the Class I GLUT family, therefore sharing certain structure similarities as, for example, the position of the glycosylation site in the extracellular loop between the transmembrane helices (TM) 1 and 2 (Joost and Thorens, 2009). For GLUT2, it has been proposed that its glycosylation is essential for proper anchoring of the transporter to the plasma membrane of β-cells and its stability in these cells (Ohtsubo et al., 2005). However, the loop itself differs in the two transporters, with GLUT2 exhibiting a significantly larger loop size than GLUT3. The role of the cytoplasmic and extracellular loops between transmembrane domains of GLUTs is still dramatically understudied and putatively underestimated. The C-terminal intracellular domain of GLUT2 has been implicated in the low glucose affinity of this transporter (Katagiri et al., 1992). Furthermore, it has been shown that conformational changes in facilitators like GLUTs, during the rocker switch mechanism, are the primary force for the translocation of sugar (Qureshi et al., 2020). Therefore, residues distant from the sugar-binding sites likely influence transport dynamics significantly, including the role of hydrophilic regions. Investigation of the extra-membrane domains will further elucidate the origins of the transporter’s different properties.

The need for a convenient platform to investigate GLUTs has been recognized, and among different systems (Gould and Lienhard, 1989; Zamora-Leon et al., 1996; Kraft et al., 2015), the yeast cell-based investigation system has many benefits (Schmidl et al., 2018). The yeast *Saccharomyces cerevisiae* is a widely used GRAS organism and a model organism for diverse research applications. Cells are easily manipulated and maintained and exhibit a short generation time. By deleting all endogenous hexose transporter genes (*HXT1-17, GAL2*) and the genes of hexose transporting maltose transporters (*AGT1, MPH2, MPH3*) with the loxP-Cre recombinase system in a CEN.PK2-1C strain background (Entian and Kötter, 2007), a hexose transporter-deficient (hxt^0^) strain, incapable of growing on glucose or related monosaccharides, was constructed and named EBY.VW4000 (Wieczorke et al., 1999; Solis-Escalante et al., 2015). The strain is maintained on maltose, a disaccharide taken up by specialized maltose symporters (Chow et al., 1989), and cleaved inside the cell into two glucose molecules. The selective uptake of the respective monosaccharide by a heterologously expressed transporter can, therefore, be examined by simple growth tests or uptake assays with the radiolabeled sugar (Boles and Oreb, 2018; Schmidl et al., 2018). Moreover, a radiolabel-free assay to determine transport kinetics in the hxt^0^ yeast system has been recently developed (Schmidl et al., 2021).

However, the functional, heterologous expression of GLUTs in this strain is a challenging task. In previous studies, the transformation of EBY.VW4000 cells with native rat GLUTs did not yield cell growth on glucose (for GLUT1 and GLUT4) (Kasahara and Kasahara, 1996; Kasahara and Kasahara, 1997) or fructose (for GLUT5) (Tripp et al., 2017). Nevertheless, single point mutations in the TM2 of GLUT1 and GLUT5 enabled their activity in EBY.VW4000 (Wieczorke et al., 2002; Tripp et al., 2017). Also, wild-type GLUT1 was functionally expressed in a hxt^0^ strain harboring the additional *fgy1* (“functional expression of GLUT1 in yeast”) mutation (Wieczorke et al., 2002) that affects the scaffold protein Efr3 (Wieczorke and Boles, personal communication). Efr3 is essential for recruiting the Stt4 phosphatidylinositol-4-kinase to the plasma membrane and, consequently, builds a prerequisite for normal membrane phosphatidylinositol-4-phosphate levels (Wu et al., 2014). The corresponding strain was named EBY.S7 (Wieczorke et al., 2002). Murine GLUT4 was only active in a strain named SDY.022 that, besides the *fgy1* mutation, had a mutation in the *ERG4* gene, coding for the terminal enzyme of the ergosterol biosynthesis pathway (Boles et al., 2004). The latter mutation putatively leads to a different sterol composition in the yeast plasma membrane, which seems beneficial for GLUT4 activity.

Here, we report the functional expression of human GLUT2 and GLUT3 in the hxt^0^ yeast system. Thereby, we complete the accessibility of the well-characterized Class I GLUTs (GLUTs1-4, categorized according to their sequence similarities (Joost and Thorens, 2009)) in a convenient system that enables detailed characterizations of these transporters and the screening for small molecules affecting their activity in a high-throughput manner. These new platforms facilitate the rapid discovery of drugs addressing severe diseases associated with these essential human transporters.

## Materials and Methods

### Strains and Media

The construction of the strains CEN.PK2-1C (Entian and Kötter, 2007), EBY.VW4000 (Wieczorke et al., 1999), EBY.S7 (Wieczorke et al., 2002) and SDY.022 (Boles et al., 2004) used in this study was reported previously and their genotypes are listed in Supplementary Table S1. For maintenance and preparation of competent cells, plasmid-free cells were grown in standard YEP-media (1% (w/v) yeast extract, 2% (w/v) peptone) supplemented with 1% (w/v) maltose. Frozen competent cells were prepared and transformed according to Gietz and Schiestl (Gietz and Schiestl, 2007). The transformants were plated on solid, selective synthetic complete (SC) medium with 1% (w/v) maltose (M) in which uracil was omitted (-URA) to maintain the selection pressure. For experiments with envyGFP constructs at the microscope, EBY.S7 or CEN.PK2-1C cells were grown in filter-sterilized, low fluorescent, synthetic complete medium (lf-SC) containing 6.9 g/l YNB with ammonium sulfate, without amino acids, without folic acid and without riboflavin (MP Biomedicals), containing 1% (w/v) maltose (for EBY.S7) or 2% (w/v) glucose (for CEN.PK2-1C) and amino acids as stated in Bruder et al. (Bruder et al., 2016), in which uracil was omitted. For subcloning of plasmids, *E. coli* strain DH10B (Gibco BRL, Gaithersburg, MD) was used.

### PCR and Plasmid Construction

DNA Sequences of GLUT2 and GLUT3 are listed in Supplementary Table S2. PCRs were performed with Phusion polymerase (New England Biolabs GmbH) and the respective primers, according to the intended modifications, which are listed in Supplementary Table S3. The resulting fragments were transformed together with the EcoR1/BamH1 linearized p426H7 vector into EBY.VW4000, EBY.S7 or SDY.022 frozen competent cells, respectively, to allow for plasmid assembly via homologous recombination (Oldenburg et al., 1997). Cells were plated on SCM (1% (w/v)) -URA agar plates and incubated for 3 days at 30°C. The grown colonies were then replica plated onto solid SC -URA medium with 0.2% (w/v) glucose (SCD (0.2% (w/v))). If growth occurred on glucose medium, single colonies from these plates were picked, sub-cultivated and plasmids were recovered by the standard alkaline lysis protocol. If no growth on glucose was observed, colonies from the maltose plates were picked and treated accordingly. For propagation and amplification, plasmids were transformed via electroporation in *E. coli*. Plasmid isolation from overnight *E. coli* cultures was carried out using a GeneJET Plasmid Miniprep Kit (Thermo Scientific) according to the manufacturer’s instructions and sequenced at GATC Biotech (Konstanz, Germany). For subcloning into a vector with a dominant marker, the respective transporter sequences were amplified from the p426H7 plasmids as described, with primers exhibiting overhangs to the *HXT7* promotor or *CYC1* terminator, respectively, and PCR products were transformed together with the linearized pRS62K plasmid, which contains the same promotor and terminator regions, into EBY.VW4000 cells to allow for homologous recombination. To check the *in vivo* localization, envyGFP (Slubowski et al., 2015) was fused to the C-terminus of transporter constructs via homologous recombination of PCR fragments presenting the specific overhangs, and the whole construct which was flanked by the *HXT7* promotor and the *CYC1* terminator was inserted into the low-copy CEN6/ARS4 vector pUCPY1. All plasmids used in this study are listed in Supplementary Table S4.

### Growth Tests

For growth tests on solid medium, drop tests were performed on minimal SC -URA medium, containing the respective sugar, with cells expressing transporter constructs in the p426H7 vector backbone. Pre-cultures were grown overnight in 10 ml SCM (1% (w/v)) -URA medium at 30°C and 180 rpm, centrifuged (3,000 g, 3 min, 20°C) and washed twice in double-distilled, sterile water (ddH_2_O). Cells were resuspended in ddH_2_O and OD_600nm_ was adjusted to 1. Dilutions of OD_600nm_ 0.1, 0.01 and 0.001 were prepared and 4 µl of each dilution was dropped onto the agar plate. Plates were incubated at 30°C for 5 days.

Cell growth in liquid YEP medium was measured with the Cell Growth Quantifier (Aquila Biolabs) (Bruder et al., 2016). Pre-cultures of cells expressing the transporter constructs in the pRS62K vector backbone were grown overnight in 10 ml YEPM (1% (w/v)) medium with 200 µg/ml G418 for plasmid selection, harvested by centrifugation (3,000 g, 3 min, 20°C) and washed twice with ddH_2_O. Washed cells were used to inoculate 30 ml YEP G418 (200 µg/ml) medium with the indicated sugar to an OD_600nm_ of 0.2 in 300 ml Erlenmeyer flasks, which were mounted onto the sensor plate. Quantification of cell growth and calculation of apparent maximal growth rates were performed with the CGQuant software (Aquila Biolabs) as previously described (Bruder et al., 2016).

### Fluorescence Microscopy

To investigate the *in vivo* localization of the (modified) transporters, CEN.PK2-1C and EBY.S7 cells expressing the envyGFP-tagged constructs on the low-copy CEN.ARS plasmid pUCPY1 were grown overnight in filter-sterilized, low fluorescent SC medium in which uracil was omitted (lf-SC -URA) and 1% (w/v) maltose (for EBY.S7 cells) or 2% (w/v) glucose (for CEN.PK2-1C) was added. 500 µl cell suspension of an OD_600nm_ between 1.5 and 3 was mixed with 500 µl lf-SC -URA medium with the respective sugar containing 1.2% (w/v) low melting agarose (Roth) to reach a suspension with 0.6% (w/v) low melting agarose for immobilization. Six microliters were applied to an object plate, sealed with a cover slip and GFP fluorescence was located with the Confocal Laser Scanning Microscope (Zeiss LSM 780, Jena, Germany).

To confirm the activity of envyGFP-tagged transporter constructs, EBY.S7 cells expressing the respective construct were streaked out on solid SC -URA medium with 0.2% (w/v) glucose and growth was recorded after 5 days of incubation at 30°C.

### Structural Modeling of GLUT2 and GLUT3

The crystal structure of GLUT2 is unknown and only the outward-facing GLUT3 structures are available. The homology models of the inward-facing GLUT2 and GLUT3 were generated with the ‘Homology Model’ function of the program package Molecular Operating Environment (MOE; Chemical Computing Group, https://www.chemcomp.com), using as a template the crystal structure of GLUT1 (PDB ID 4PYP). The homology models for the outward-facing GLUT2 were generated with MOE from the crystal structure of GLUT3 (PDB ID 5C65 or 4ZWC). The amino acid sequence identity and similarity between GLUT2 and GLUT3 are 50% and 68%, between GLUT1 and GLUT2 are 52% and 68%, and between GLUT1 and GLUT3 are 63% and 78%, respectively, as determined with the alignment function from MOE. The homology models generated were scored with GB/VI. The mutations were performed in MOE Protein Designing function and subject to energy minimization with the Forcefield Amber10.

### Transport Assay for Inhibition Studies

The culturing of yeast cells was done at 30°C with shaking (180 rpm). EBY.S7 yeast cells expressing GLUT2_∆loopS_Q455R_ were grown for a day in YEPM (1% (w/v)) media containing 200 µg/ml G418. Cells were washed once in YEP media containing 0.2% (w/v) glucose (YEPG) and 200 µg/ml G418 and transferred in the same media so that OD_600nm_ ∼ 0.5 and grown further for 1 to 2 days. GLUT3_S66Y_ expressed in EBY.S7 were grown for two days in SC-URA with 1% (w/v) maltose, then washed and transferred in SC-URA with 0.2% (w/v) glucose, followed by further growth for 1-2 days. For transport activity assay, cells were centrifuged (1000 g, 5 min), washed once with PBS solution (10 mM Na_2_HPO_4_, 1.8 mM KH_2_PO_4_, 2.7 mM KCl, 137 mM NaCl, pH 7.4), and resuspended in PBS buffer at an OD_600nm_ ∼ 10; each assay determination contained 100 µl of this cell solution. Transport activity assay was started by adding C^14^-glucose (10 mM for GLUT2_∆loopS_Q455R_ or 1 mM for GLUT3_S66Y_). Transport activity assay was halted after 10 min by adding 3 ml ice-chilled Quench buffer (0.1 M KPi, 0.1 M LiCl, pH 5.5), followed by filtration through a glass fiber channel (GC50; Advantec, Tokyo, Japan) under vacuum, and another two washes with 3 ml Quench buffer and filtration. The filtration membranes were transferred into scintillation vials with 10 ml of Scintillation Solution (BioSafeII; Research Products International, Mount Prospect, IL, United States), and vortexed briefly. The radioactivity was determined with a scintillation counter (Tri-carb 2900TR, Perkin Elmer, Waltham, MA, USA). Phloretin and quercetin were dissolved in DMSO at 20 mM stock concentration, and inhibitor concentrations used for IC_50_ determination were 100x stocks so that the final concentration of DMSO in the assay was 1%. Controls for determining the relative transport activity included 1% (v/v) DMSO to account for DMSO presence due to inhibitor, and the cells transformed with the empty vector. We found that the background activity with the empty vector was comparable to the transport activity of the transporters at 200 µM phloretin. Data were analyzed with the nonlinear fit analysis of GraphPad Prism (San Diego, CA, United States).

## Results

### Generation of GLUT Constructs that Mediate Glucose Uptake into the hxt^0^ Yeast Strain

#### A GLUT3 Mutant Shows Enhanced Activity in the hxt^0^ Yeast System

**FIGURE 1:**
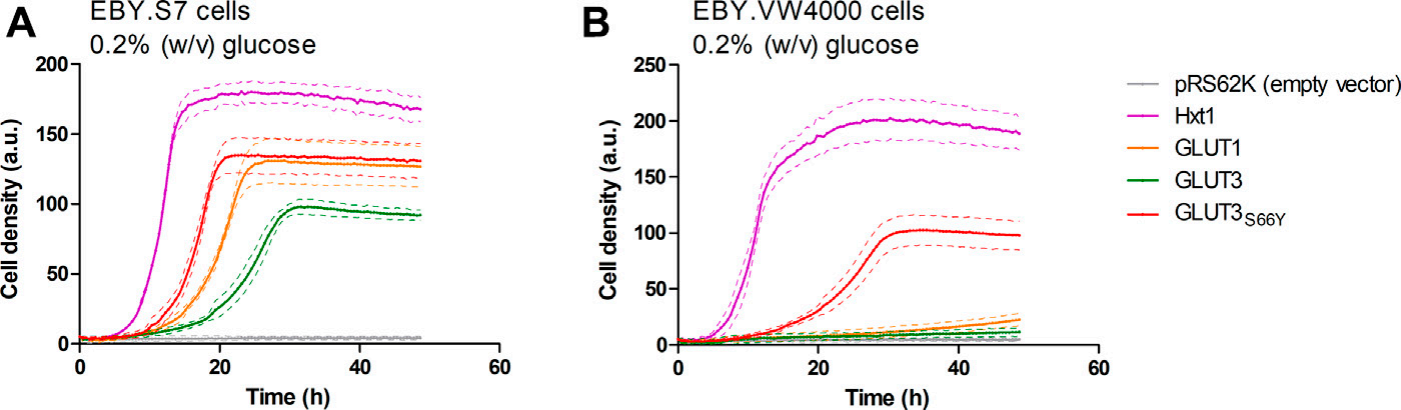
Growth of hxt^0^ cells expressing GLUT3 or GLUT3_S66Y_ on glucose. Cell growth of EBY.S7 **(A)** or EBY.VW4000 **(B)** cells in the YEP medium containing 200 µg/ml G418 and 0.2% (w/v) glucose was recorded with the Cell Growth Quantifier (Aquila Biolabs). The cells were transformed with the empty pRS62K vector, pRS62K vector or with plasmids encoding the endogenous Hxt1 the endogenous Hxt1 transporter, GLUT1, GLUT3, or GLUT3_S66Y_. The average of three biological replicates (continuous lines) and the error (dashed lines) are shown. Apparent maximal growth rates for the growing cells were calculated with the CGQuant software and are listed together with the duration of lag phases in Supplementary Table S5.

A plasmid for the expression of GLUT3 in yeast was generated using either the native human coding sequence or a codon-optimized sequence for the expression in insect cells. Amplification of the open reading frame (ORF) with oligonucleotides having 30-40 base pair overhangs to the applied promoter (*HXT7* ^-1–329^) or terminator (*CYC1*) region, respectively, and co-transformation with the linearized host plasmid (p426H7) in EBY.VW4000, EBY.S7, and SDY.022 cells allowed for the plasmid assembly via homologous recombination (Oldenburg et al., 1997; Boles and Oreb, 2018). The strong, truncated *HXT7* promoter region and the *CYC1* terminator were chosen to achieve high expression levels. Transformants were plated on selective SC -URA medium, containing 1% (w/v) maltose, and incubated for three days at 30°C, resulting in the growth of approximately 1000 colonies per plate. Subsequently, cells were replica plated on SC -URA medium, containing 0.2% (w/v) glucose, to screen for cells that regained the ability to use glucose as a carbon source. EBY.VW4000 transformants did not show any growth on the solid glucose medium, even after a prolonged incubation of seven days. Most of the EBY.S7 and SDY.022 transformants grew after five days of incubation on glucose. To investigate if wild-type GLUT3 is active in these strain backgrounds, we isolated and sequenced plasmids of several colonies with various sizes. In bigger colonies, one single mutation (S66Y) was found, located in TM2. However, also unmodified GLUT3 mediated the growth of EBY.S7 and SDY.022 transformants on glucose. Re-transformation of the native GLUT3 and the modified GLUT3_S66Y_ construct in EBY.S7 cells resulted in growth on glucose medium, proving that no further mutations, except for the *fgy1* mutation, in the yeast strain were essential for this phenotype. However, while wild-type GLUT3 was incapable of mediating growth of EBY.VW4000 cells on solid glucose medium, slight growth was observed for GLUT3_S66Y_-expressing EBY.VW4000 cells (Supplementary Figure S7A). Furthermore, the drop test of the EBY.S7 and SDY.022 cells expressing the different constructs (GLUT3 and GLUT3_S66Y_) on a plasmid, revealed larger colonies for those expressing the mutated version (Supplementary Figures S7B,C), indicating that the S66Y mutation is beneficial for the functionality of the transporter in the heterologous system. Growth tests with hxt^0^ cells expressing GLUT3 or GLUT3_S66Y_ were performed in liquid YEP medium with 0.2% (w/v) glucose, for which the GLUT3 ORFs were transferred to pRS62K plasmid backbones. Consistent with the results observed in the drop tests, both constructs enabled the growth of EBY.S7 cells, but GLUT3-expressing cells showed a significantly longer lag phase and a 1.8 times lower apparent maximal growth rate than those expressing GLUT3_S66Y_ (Figure 1A; Supplementary Table S5). For EBY.VW4000 cells, only GLUT3_S66Y_ mediated growth, while the unmodified GLUT3 did not (Figure 1B). Positive controls were GLUT1, as a representative of a heterologous transporter active in EBY.S7 cells (Wieczorke et al., 2002), and Hxt1, an endogenous low-affinity transporter for glucose and fructose (Leandro et al., 2009).

#### Site-Directed Mutagenesis Approaches to Enable GLUT2 Activity in Yeast

Expression plasmids carrying either the native or a codon-optimized (for insect cells) human GLUT2 sequence were generated as described above for GLUT3. However, after replica plating, EBY.VW4000, EBY.S7, or SDY.022 transformants, of which more than 1000 colonies grew on solid maltose medium, showed no growth on glucose-containing agar plates, even after prolonged incubation at 30°C for one week.

Like the S66Y mutation in GLUT3, previous studies found single point mutations in the second transmembrane region of GLUT1 (e.g., V69M) (Wieczorke et al., 2002) and GLUT5 (e.g., S72Y and S76I) (Tripp et al., 2017) that enabled or improved the functional expression of these GLUTs in yeast. Therefore, we focused on this critical region in GLUT2 as well. Comparison of the amino acids 96 – 104 in GLUT2 with the corresponding area in GLUT1, GLUT3, and GLUT5 (Supplementary Figure S2) revealed that GLUT2 primary sequence has two consecutive serines at positions 102-103 vs. hydrophobic amino acids in the other GLUTs successfully expressed in yeast (Supplementary Figure S2).

Interestingly, in GLUT3 and GLUT5, a single serine mutation to a more hydrophobic amino acid enabled or improved the functional expression of these two transporters in yeast (as shown in this study and by Tripp et al. (Tripp et al., 2017)). This observation prompted us to direct mutagenesis to the amino acids 101-103, including the valine at position 101, considering the effect of the V69M mutation in GLUT1 (Wieczorke et al., 2002). By using degenerate primers that allow for the introduction of different nucleotides at a particular position (Kwok et al., 1994) (see Supplementary Table S3), different codons were inserted to mutate the chosen amino acids. The following degenerate codons were used to insert the desired range of substitutions: at position 101, RTK (encoding Ile, Met and Val); at position 102, RYT (encoding Ala, Ile, Thr and Val); at position 103, ATK (encoding Ile and Met). Considering all permutations, a library encoding 24 different protein sequence variants of the critical region resulted from this approach. The sequencing of the isolated plasmids from random clones confirmed the successful introduction of the expected range of substitutions. Still, screening of more than 1000 colonies for growth on glucose-containing agar plates yielded no positive clones. In addition to these mutations in TM2, we mutated the N glycosylation site at position 62 (GLUT2_N62Q_) within the first extracellular loop, just in case the glycosylation disturbed the activity of the transporter in yeast cells. This modification also failed to restore hxt^0^ yeast cell growth on glucose.

#### Modification of the Extended Extracellular Loop of GLUT2

**FIGURE 2:**
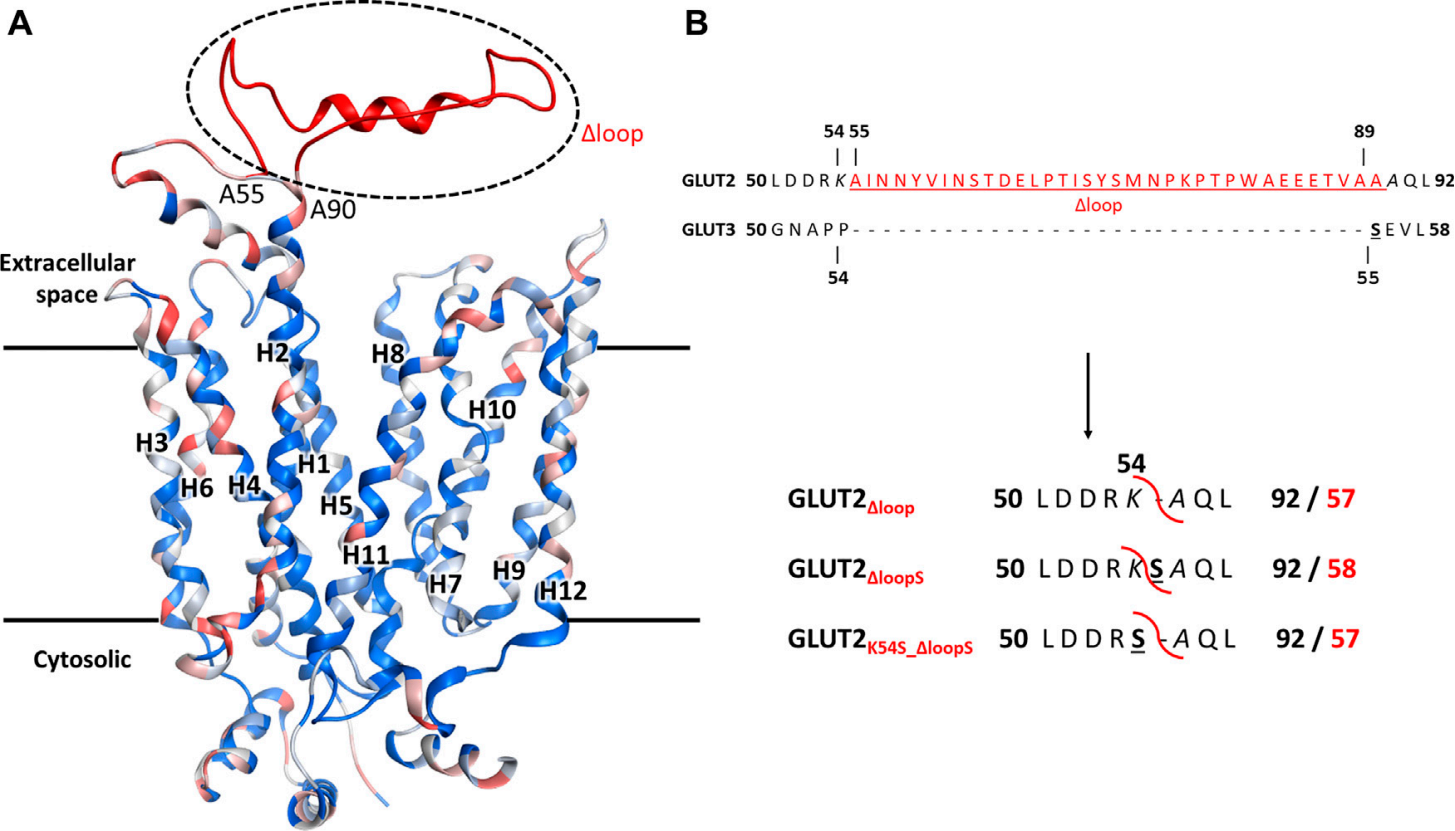
The structural model for GLUT2, GLUT3, and GLUT2 constructs. **(A)** The structural model was generated in MOE, based on the crystal structure of the outward-facing conformation of human GLUT3 (PDB ID 4ZWC). The color code represents the sequence homology between GLUT2 and GLUT3, ranging from identical residues (dark blue) to very dissimilar substitutions (red). Unique to GLUT2 is the extension in the loop between transmembrane helices H1 and H2, in the extracellular space (shown in red). **(B)** GLUT2 and GLUT3 sequence alignment in the region flanking the extended extracellular loop of GLUT2 (in red, underlined). The loop deletion constructs of GLUT2 are: GLUT2_Δloop_, which lacks residues 55-89, GLUT2_ΔloopS_, which lacks residues 55-88 and has the mutation A89S, and GLUT2_K54S_Δloop_, which lacks residues 55-89 and has the mutation K54S. For the two latter constructs, the single substitutions to serine are in analogy to S55 of GLUT3 (black bold underlined). The red curved line in the constructs shows the loop excision site. For the GLUT2 constructs, the number for both the original (bold black) and post-deletion (bold red) sequences are shown.

A conspicuous structural difference between GLUT2 and the other Class I GLUTs, which are active in yeast, is the extension of the extracellular loop connecting the TM helices H1 and H2 (Figure 2).

Since minor sequence modifications have not led to an active expression of GLUT2 in hxt^0^ yeast strains, we reasoned that shortening the extension, thereby introducing a GLUT3-like-loop, could promote GLUT2 activity in the heterologous system.

In addition to just deleting the amino acids 55-89 (GLUT2_∆loop_), we also constructed one chimera in which those amino acids were replaced by one serine (GLUT2_∆loopS_) and one in which the lysine preceding the deleted region was mutated to serine (GLUT2_K54S_∆loop_) (Figure 2B). Compared to GLUT3, the loop of GLUT2_∆loop_ is one residue shorter, lacking the residue corresponding to S55 of GLUT3, while that of GLUT2_∆loopS_ is the same length and includes a residue in analogy to S55 of GLUT3. GLUT2_K54S_∆loop_ contains the mutation corresponding to S55 of GLUT3 but at the beginning instead of the end of the deleted loop. The modifications were introduced into the ORFs via PCR (primers are listed in Supplementary Table S3), and the individual fragments were transformed together with a linearized p426H7 vector in EBY.VW4000, EBY.S7, or SDY.022 cells. Transformants that grew on solid maltose medium (more than 1000 colonies per plate) were then replica plated onto glucose containing plates. A substantial number (approximately 60%) of colonies expressing one of the three loop-modified constructs, respectively, grew on glucose. Multiple plasmids from each strain background and loop-construct combination were isolated and sequenced. Most of these plasmids did not show mutations other than those intentionally introduced. Strikingly, in 2 out of 3 sequenced plasmids harboring the GLUT2_∆loopS_ construct that were isolated from EBY.VW4000 cells, a point mutation led to the substitution of the amino acid glutamine at position 455 with either lysine (Q455K) or arginine (Q455R). Also, Q455R mutation was found in one out of three GLUT2_∆loopS_ constructs isolated from SDY.022 cells, indicating an additional beneficial effect of this mutation on the glucose uptake via GLUT2. The isolated loop constructs GLUT2_∆loop_, GLUT2_K55S_∆loop_, GLUT2_∆loopS_, and GLUT2_∆loopS_Q455R_ were re-transformed in all three hxt^0^ strains, and their growth was analyzed by a drop test on solid glucose medium (Supplementary Figure S9). The ability to grow on glucose was regained by EBY.S7 and SDY.022 cells with all loop constructs, whereas cells expressing GLUT2_∆loopS_ and especially GLUT2_∆loopS_Q455R_, respectively, showed bigger colonies in comparison to GLUT2_∆loop_ or GLUT2_K55S_∆loop_ expressing cells (Supplementary Figures S9B,C). EBY.VW4000 cells grew significantly only when expressing GLUT2_∆loopS_Q455R_ (Supplementary Figure S9), providing further evidence for a crucial effect of this additional point mutation, when combined with the ∆loopS modification, on GLUT2 activity.

**FIGURE 3:**
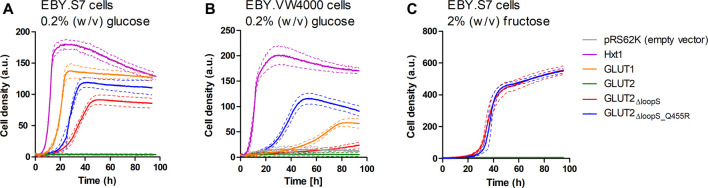
Growth of hxt^0^ cells expressing GLUT2 variants on hexoses. Cell growth of EBY.S7 **(A,C)** or EBY.VW4000 **(B)** cells containing the empty pRS62K vector, the endogenous Hxt1 transporter (only **(A)**,**(B)**), GLUT1 (only **(A)**,**(B)**), GLUT2, GLUT2_ΔloopS_ or GLUT2_ΔloopS_Q455R_, was recorded with the Cell Growth Quantifier (Aquila Biolabs). The YEP medium was supplemented with 200 μg/ml G418 for plasmid selection and 0.2% (w/v) glucose **(A,B)** or 2% (w/v) fructose **(C)**. The average of three biological replicates (continuous lines) and the error (dashed lines) are shown. Apparent maximal growth rates for the growing cells were calculated with the CGQuant software and are listed together with the duration of lag phases in Supplementary Table S5.

As with GLUT3, the ORFs of different GLUT2 variants were transferred into the pRS62K vector and tested for growth in liquid YEP media, using the Cell Growth Quantifier (Aquila Biolabs) (Bruder et al., 2016). In agreement with the results from the drop test, only GLUT2_∆loopS_ and GLUT2_∆loopS_Q455R_, but not wild-type GLUT2, conferred growth of EBY.S7 cells on 0.2% (w/v) glucose (Figure 3A). For EBY.VW4000 cells, the additional point mutation in GLUT2_∆loopS_Q455R_ was essential for growth in this medium (Figure 3B). Interestingly, no growth of EBY.S7 cells expressing either of the loop constructs was observed on 0.2% (w/v) fructose medium, while the positive controls expressing Hxt1 showed normal growth (data not shown). This is likely due to the very low affinity of GLUT2 for fructose (*K*_*M*_ = ∼76 mM) (Mueckler and Thorens, 2013). However, on 2% (w/v) fructose, both loop constructs conferred robust growth (Figure 3C). Analysis of the apparent maximal growth rates with the CGQuant Software (Bruder et al., 2016) showed a significantly faster doubling rate for EBY.S7 cells expressing GLUT2_∆loopS_Q455R_ compared to GLUT2_∆loopS_-expressing cells on 0.2% (w/v) glucose medium (1.7 times) as well as on 2% (w/v) fructose medium (1.5 times) (Supplementary Table S5).

### Subcellular Localization of Different GLUT2 Variants

**FIGURE 4:**
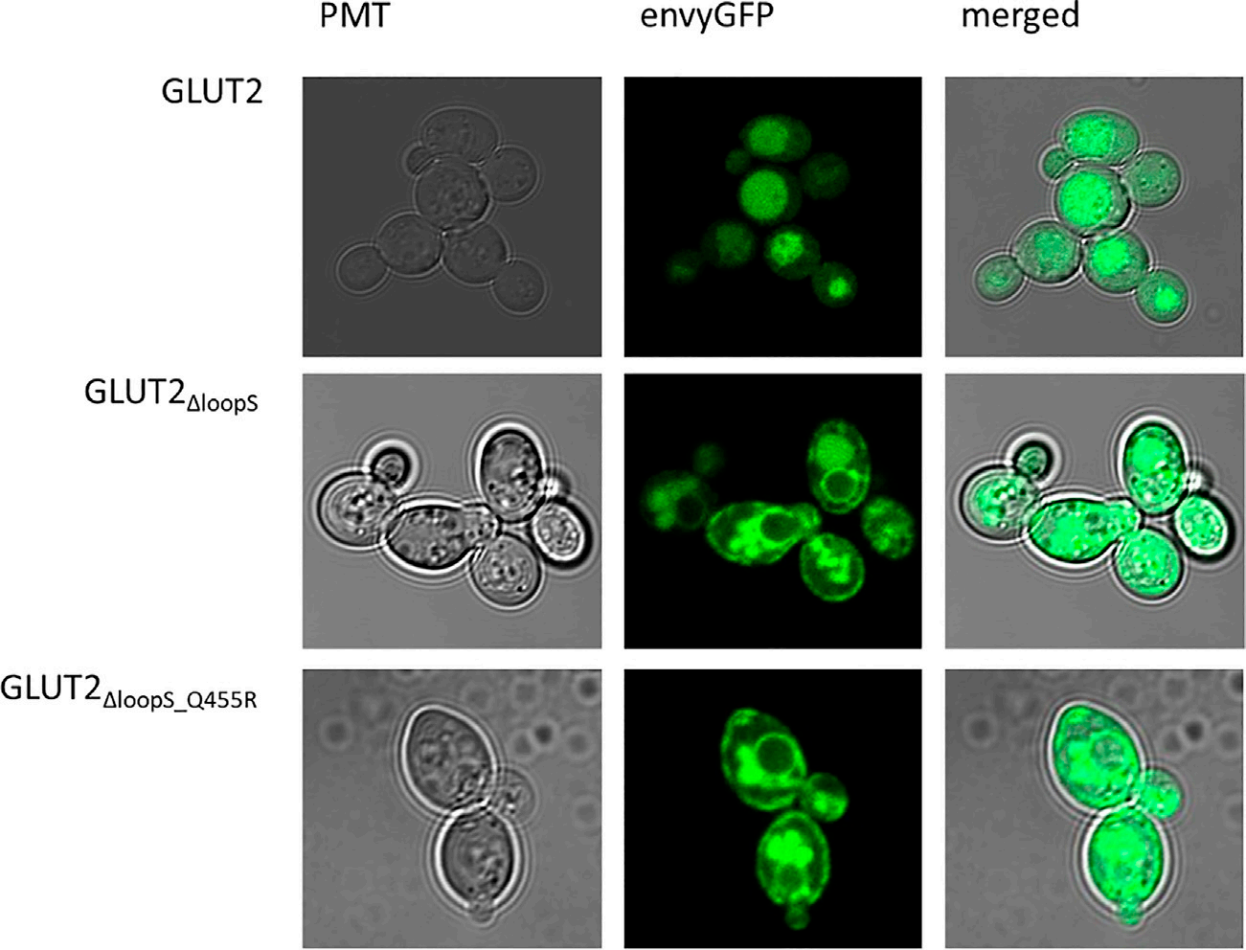
Subcellular localization of GLUT2 variants. envyGFP was fused to the C-termini of GLUT2 (wild-type), GLUT2_ΔloopS_, and GLUT2_ΔloopS_Q455R_. CEN.PK2-1C cells, expressing the respective envyGFP construct, were grown in low fluorescent SC -URA medium containing 0.2% (w/v) glucose. For immobilization, 0.6% (w/v) low melt agarose was added to the suspension. Localization was analyzed with the Confocal Laser Scanning Microscope (Zeiss LSM 780, Jena, Germany).

One hypothesis to explain the adverse effect of the extended loop on GLUT2 activity in yeast is possible interference with the transporter’s targeting to the plasma membrane. To test if our loop modifications influence the transporter trafficking, we fused the strong GFP variant envyGFP (Slubowski et al., 2015) to the C-terminal ends of the two most active constructs GLUT2_∆loopS_ and GLUT2_∆loopS_Q455R_, and of the inactive wild-type GLUT2. The activity of these envyGFP-tagged transporters was confirmed by the growth of GLUT2_∆loopS_Q455R_-envyGFP and GLUT2_∆loopS_-envyGFP-expressing EBY.S7 cells on solid SC -URA 0.2% (w/v) glucose medium (Supplementary Figure S11). Fluorescence microscopy of CEN.PK2-1C cells indeed revealed significant differences between the localization of the wild-type GLUT2 and the two truncated constructs. While cells expressing the latter showed a patchy distribution at the plasma membrane and partial retention in the endomembrane system, unmodified GLUT2 does not reach the plasma membrane and is located in vacuoles (Figure 4), thereby being destined for degradation.

The same pattern was observed in EBY.S7 cells (Supplementary Figure S10), demonstrating that the effect of the loop is not dependent on the strain background. Therefore, we conclude that the implemented loop modifications rescue the trafficking defect of GLUT2 in yeast cells. The additional point mutation appears not to influence the subcellular localization of the transporter and putatively improves the transporter’s substrate-gating activity by other effects (see below).

### The Yeast Platform is Suitable for Screening and Characterizing GLUT Inhibitors

**FIGURE 5:**
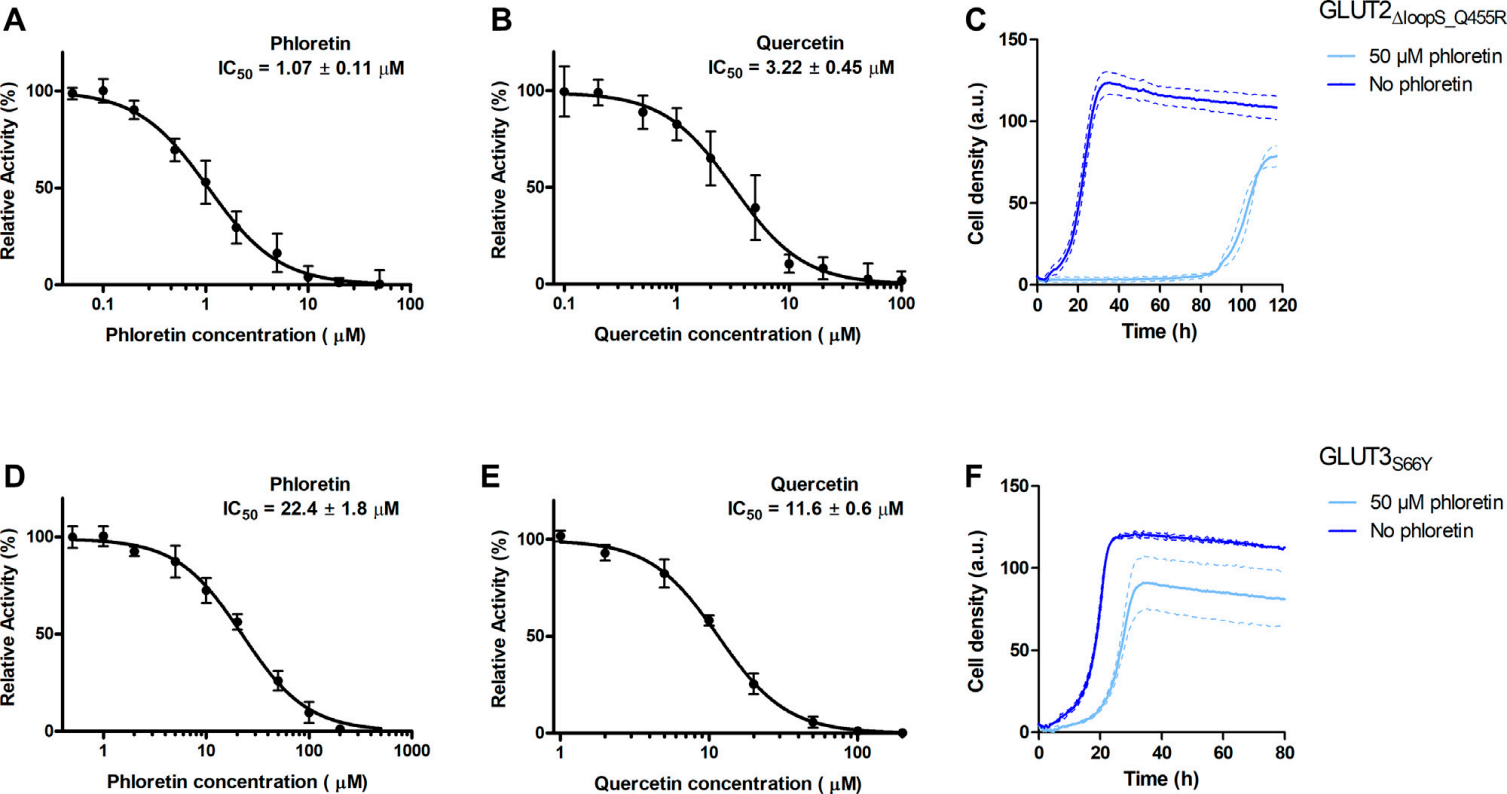
Effect of phloretin and quercetin on GLUT2_∆loopS_Q455R_ and GLUT3_S66Y_ expressed in hxt^0^ EBY.S7 cells. The relative transport activity (expressed as % of the transport in the absence of the inhibitor) of GLUT2_∆loopS_Q455R_ **(A,B)** and GLUT3_S66Y_ **(D,E)** as a function of phloretin **(A,D)** or quercetin **(B,E)** concentrations at 10 mM (for GLUT2_∆loopS_Q455R_, **(A,B)**) or 1 mM (for GLUT3_S66Y_, **(D,E)**) glucose is shown. Error bars represent the standard deviation from three independent measurements. Growth assays with GLUT2_∆loopS_Q455R_ **(C)** or GLUT3_S66Y_ **(F)** expressing EBY.S7 cells on YEP medium supplemented with 0.2% (w/v) glucose and G418 (200 μg/ml) in the presence of 50 μM phloretin (light blue) or its absence (dark blue) were recorded with the Cell Growth Quantifier (Aquila Biolabs). The average of two biological replicates (continuous lines) and the error (dashed lines) are shown. Apparent maximal growth rates of the here-depicted experiments, and EBY.S7 cells expressing Hxt1 with and without phloretin as a control, were calculated with the CGQuant software and are listed together with the duration of lag phases in Supplementary Table S6.

Since the best activities of GLUT2 and GLUT3 constructs were observed in the EBY.S7 cells, this strain background was chosen to establish a yeast-based platform for screening and characterizing GLUT inhibitors. Phloretin (Kwon et al., 2007) and quercetin (Lee et al., 2015) are well-known inhibitors of GLUT2 and GLUT3 (Kwon et al., 2007; Augustin, 2010; Lee et al., 2015), and, therefore, we determined their half maximal inhibitory concentration (IC_50_ values) for GLUT2_∆loopS_Q455R_ expressed in EBY.S7 (Figures 5A,B).

The values obtained for GLUT2_∆loopS_Q455R_ (IC_50_ of 1.07 ± 0.11 and 3.22 ± 0.45 µM for phloretin and quercetin, respectively) indicate a strong inhibition and confirm that yeast-expressed GLUT2 can be reliably used to identify and characterize transport inhibitors despite the deletion of the extended extracellular loop. This is supported by growth tests in the presence of phloretin. We found that 50 µM phloretin inhibited the growth of EBY.S7 cells expressing GLUT2_∆loopS_Q455R_ significantly, leading to an extensive long lag phase of 87.4 hours, a diminished apparent maximal growth rate (Supplementary Table S6) and a lower maximal cell density (Figure 5C).

Inhibition of GLUT3_S66Y_ with phloretin and quercetin is less potent than for GLUT2_∆loopS_Q455R_ but still effective, as demonstrated by the IC_50_ values (22.4 ± 1.8 µM for phloretin and 11.6 ± 0.6 µM for quercetin) (Figures 5D,E). Moreover, the phloretin IC_50_ values are in the same range as those reported for the related GLUT1 and GLUT4 (Kasahara and Kasahara, 1997). Reflecting the higher IC_50_ values of phloretin compared to those measured with GLUT2_∆loopS_Q455R_, the growth delay of GLUT3_S66Y_ expressing EBY.S7 cells in glucose containing medium in the presence of 50 µM phloretin is less pronounced but still significant (Figure 5F; Supplementary Table S6). In contrast, the apparent maximal growth rates of Hxt1-expressing EBY.S7 cells do not show significant differences when grown in the absence or presence of 50 μM phloretin (Supplementary Table S6). As all tested transporters were expressed under the control of the truncated Hxt7 promotor, the Hxt1 control shows that the diminished growth phenotypes of GLUT2_∆loopS_Q455R_ and GLUT3_S66Y_-expressing EBY.S7 cells upon phloretin addition do not arise from lowered expression levels. Together, these data demonstrate that the yeast system can be conveniently used to screen large compound libraries for potential inhibitors of GLUT2 and GLUT3 in a simple, growth-based manner, amenable to high throughput screening.

### Structural Analysis of GLUT2 and GLUT3 Constructs

**FIGURE 6:**
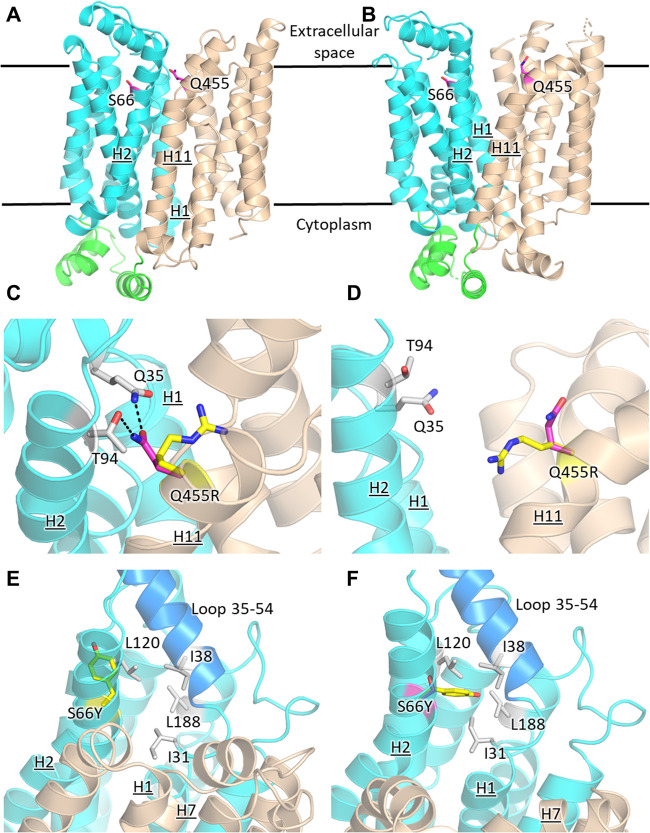
Structural models of GLUT2 and GLUT3 variants with functional transporter expression in hxt^0^ cells. Inward-facing **(A,C,E)** and outward-facing **(B,D,F)** conformations of GLUT2 and GLUT3 based on the crystal structures for the corresponding conformations of GLUT1 (PDB ID 4PYP, inward-facing) or GLUT3 (PDB ID 5C65, outward-facing), showing the locations of the single-site mutations that increased the transport activity of GLUT2_loop_ and GLUT3 expressed in hxt^0^ yeast cells: S66 in H2 of GLUT3 and Q455 in H11 of GLUT2_loop_. The transporter N- and C-halves are in magenta and gold, respectively, and the transmembrane helices are labeled as H1-H12. **(C,D)** Close-up views of **(A,B)**, respectively, focused on Q455R in GLUT2_loop_, showing the original side-chain (magenta), and the mutant (yellow). **(E,F)** Close-up views of **(A,B)**, respectively, focused on S66Y in GLUT3. The mutant side chain is shown in yellow. The extracellular loop between helices H1 and H2 (residues 35-54, shown in blue) is close to the mutated side chain Y66 in the outward-facing conformation **(F)**. Compared with the inward-facing conformation, in the outward-facing conformation, the loop spanning residues 35-54 moves at least 1 Å in the direction indicated by the red arrow. Figures were drawn using Open-Source PyMol Version 2.3.0 (The PyMOL Molecular Graphics System, Version 2.3.0 Schrödinger, LLC).

The prevailing transport mechanism for the Major Facilitator Superfamily (MFS) proteins, including GLUTs, postulates that the substrate cavity of the transporter is alternately open to either side of the membrane with the transporter cycling between the so-called outward- and inward-facing conformations, through a relative rocking of the transporter N- and C- halves (domains) around the active site (Abramson et al., 2003; Yan, 2015). The structural models for GLUT2_ΔloopS_, GLUT2_ΔloopS_Q455R_, GLUT3, and GLUT3_S66Y_ were based on the crystal structures of the inward-facing conformation for GLUT1 (PDB ID 4PYP) and the outward-facing conformation of GLUT3 (PDB ID 5C65 or 4ZWC) (Figure 6).

GLUT2 and GLUT3 share 50% and 68% amino acid sequence identity and homology, respectively (determined with MOE, Chemical Computing Group). Cell growth of GLUT-expressing EBY.S7 cells showed that additional single mutations in GLUT2_ΔloopS_ and GLUT3 improved the transport activities (Figures 1, 3): Q455R in GLUT2_ΔloopS_ and S66Y in GLUT3. Q455 of GLUT2 is in the outward edge of the TM helix H11, close to the interface between the N- and C-domains (Figure 6A). Its substitution with arginine is inconsequential for the outward-facing conformation (Figures 6B,D), but disruptive for the inward-facing conformation of the transporter; the bulkier guanidinium moiety can no longer participate in the hydrogen bond interactions with Q35 of H1 and T94 of H2 (Figure 6C), sterically crowding its vicinity. In GLUT3, S66 is in the outer periphery of H2, part of a hydrophobic pocket composed of N-domain residues including, I31, I38, L120, and L188, close to the soluble loop linking H1 and H2 (Figures 6A,E,F). In the outward-facing conformation of GLUT3, S66 substitution with the larger side-chain of a tyrosine integrates well, as there is ample space around the loop connecting H1 and H2 (loop 35-54) (Figure 6F). Conversely, in the inward-facing conformation, loop 35-54 packs near H7, and the tyrosine side-chain is pushed away from the hydrophobic pocket (Figure 6E).

## Discussion

The work here completes the generation of GLUT-specific assay systems for class I GLUTs in the versatile and simple platform provided by the engineered yeast cells, hxt^0^. These systems enable high-throughput ligand screening for a particular GLUT and determination of the potency and selectivity of the ligand candidates relative to closely related GLUTs within class I, and also class II member, GLUT5. The co-existence of several GLUT isoforms within one tissue or cancer cell line complicates efforts to understand the role of a particular GLUT in the cell pathology or physiology. Thus, besides their medical importance as potential drugs, GLUT-selective inhibitors can also serve as investigative tools to unravel how healthy and abnormal cells adjust their energetic needs through GLUT expression, regulation or function (Carreño et al., 2019). Additionally, GLUT-specific substrate analogs can be optimized as diagnostic tools, especially in cancer detection (Barron et al., 2016; George Thompson et al., 2016; Wuest et al., 2018). Finally, discovery of GLUT-selective activators would be of particular interest in the case of GLUT4 or GLUT2, as strategies to ameliorate diabetes.

Having a GLUT2 assay system is particularly useful. The low affinity for glucose and fructose of this transporter makes it difficult to study its activity when other GLUT isoforms are present, even when GLUT2 is overexpressed. Deleting the loop extension between TM1 and TM2 helices was necessary for the functional expression of GLUT2, and the two transporters showed differences in their inhibition by phloretin and quercetin, with GLUT2 being significantly more sensitive than GLUT3 (Figure 5).

**FIGURE 7:**
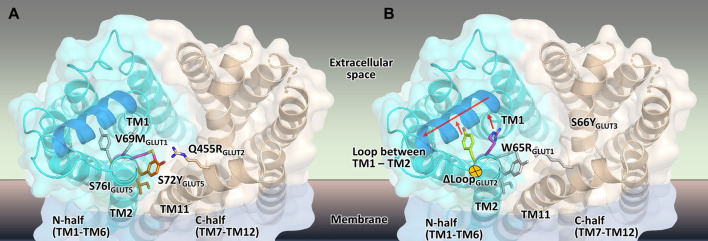
Mapping the mutations that render different GLUTs active in hxt^0^ yeast cells. **(A,B)** The structural model of the outward-facing conformation for GLUT1-3 and GLUT5 showing the single-site mutations that provide or increase transport activity of the respective human GLUT upon expression in hxt^0^ yeast cells, viewed from the extracellular side. The mutants are generally substitutions to bulkier side chains and fall into two categories. **(A)** Mutants that disrupt the packing between the N- and C-domains (colored as cyan and wheat, respectively), such as S72I and S76Y of GLUT5, Q455R of GLUT2, and V69M of GLUT1. **(B)** Mutants that dislocate the loop between TM helices TM1 and TM2 (the part of the loop that needs to move to accommodate the mutations is shown in blue), causing it to move away from the central cavity, such as S66Y of GLUT3 and W65R of GLUT1.

Besides their value for GLUT-specific ligand discovery and characterization, the GLUT expressing yeast systems point to intriguing clues regarding the regions important for transporters activity. As shown here for GLUT2 and GLUT3 and previously reported for GLUT1 (Wieczorke et al., 2002) and GLUT5 (Tripp et al., 2017), the functional expression of these transporters is facilitated or improved by single amino acid substitutions in the transporter sequence (summarized in Table 1, Figure 7). Although the elucidation of the underlying mechanism is complex and not in the scope of this study, structural modeling can help derive hypotheses to explain the observations.

**TABLE 1:** Mutations that enable the transport activity of human GLUTs expressed in hxt^0^ cells.

Protein	Mutation	Reference
GLUT1	W65R	Wieczorke et al. (2002)
	V69M	
GLUT2_ΔloopS_	Q455R	This study
GLUT3	S66Y	This study
GLUT	S72Y	Tripp et al. (2017)
	S76I	

Wild-type transporters and GLUT2_ΔloopS_, although localized at the membrane, could not compensate for the absence of the endogenous hexose transporters when EBY.VW4000 cells were grown in media with glucose or fructose as the carbon source. W65R and V69M of GLUT1 (Wieczorke et al., 2002), S66Y of GLUT3 (this study), S72Y and S76I of GLUT5 (Tripp et al., 2017) are all residues in the TM2, near the loop connecting TM helices 1 and 2, which in turn either packs closely to TM helices 7 and 11 (in the inward-facing conformation) or relaxes by moving at least 1 Å away from these helices (in the outward-facing conformation). Intriguingly, Q455R of GLUT2_ΔloopS_ is at the end of TM11, which also approaches the same region: TM1, TM2, and their connecting loop (residues 35-54) (Figures 6, 7). The convergence in the location of the mutations that functionalize the transporters (Figure 7), whether they are part of the Class I GLUTs (like GLUT1-3) or Class II (like GLUT5), suggests that this region is critical for transport activity of human GLUTs in the yeast system. Consistent with the proposed role of the mutations in GLUT5 (Tripp et al., 2017), the ones described here for GLUT3 and GLUT2_ΔloopS_ (S66Y and Q455R, respectively) also seem to destabilize the inward-facing conformation while leaving the outward-facing one unaffected. More generally, the substitutions that functionalize GLUTs in yeast cells favor an open substrate cavity towards the extracellular space (i.e., outward-facing conformation), either by destabilizing the N- and C-domain interface of the inward-facing conformation (Figure 7A) or by pushing the TM1-TM2 extracellular loop away, leading to a more accessible substrate cavity from the extracellular side (Figure 7B).

Evidently, the lipid composition of the yeast’s plasma membrane also impacts the heterologous expression of some human GLUTs (Wieczorke et al., 2002; Schmidl et al., 2018).

In EBY.S7, a strain in which the phosphatidylinositol-4-phosphate composition is putatively altered due to the *fgy1* mutation (Schmidl et al., 2018), wild-type GLUT1 and GLUT3, and GLUT2_ΔloopS_, are actively expressed without any further point mutations. Conceivably, the cycling between the transporter conformations depends on the lipid environment; the relative stabilization of the outward-facing conformation vs. the inward-facing one may be a feature of functional expression of human GLUTs in yeast cells. Activating transport by stabilizing the outward-facing conformation has been documented for another classic MFS protein, LacY, for which interaction of the transporter periplasmic loops with a nanobody against the outward-facing conformation increased the substrate affinity 50-fold (Smirnova et al., 2014). Strikingly, the critical role of lipids in yeast for the functionality of transporters that undergo conformational changes during the transport cycle has been recently demonstrated, supporting our hypothesis (van’t Klooster et al., 2020a; van’t Klooster et al., 2020b).

For GLUT2, introducing point mutations or screening a combinatorial mini-library of 24 possible aminoacid combinations in the critical TM2 region was not sufficient for activating the transporter, even in strain backgrounds EBY.S7 and SDY.022 exhibiting an altered lipid composition. We demonstrated that truncating the large extension of the second extracellular loop, which is a distinctive property of GLUT2 compared to other GLUTs functionally expressed in yeast, was a successful strategy based on a rational approach. As shown by fluorescence microscopy, this modification promotes the trafficking of the transporter to the plasma membrane, whereas the full-length protein is largely retained in the vacuole. In general, the role of the soluble loops in MFS transporters is not well understood, but it is believed that they might affect the gating and conformational dynamics of the transporters (Qureshi et al., 2020). Our results suggest that the GLUT2-specific extension of the second loop is not essential for glucose transport per se. It might rather have a regulatory function, which is probably relevant only within its native environment in the human cells. Similar to our observations, the soluble loops were dispensable for the function of the human nucleoside transporters (belonging to the MFS superfamily) expressed in *Xenopus* oocytes (Aseervatham et al., 2015).

In summary, despite the necessary modifications of their sequence, our transporter variants retain their native function in facilitating glucose (GLUT2, GLUT3) and fructose (GLUT2) uptake. Importantly, they are sensitive to the known inhibitors phloretin and quercetin, which validates our system as a platform for screening and characterizing potential transport inhibitors.

## Data Availability

The raw data supporting the conclusions of this article will be made available by the authors, without undue reservation.
