# Supplementary material for: Functional Expression of the Human Glucose Transporters GLUT2 and GLUT3 in Yeast Offers Novel Screening Systems for GLUT-Targeting Drugs
Source: Front Mol Biosci. 2021 Feb 18;7:598419. doi: 10.3389/fmolb.2020.598419 (PMC7930720; doi:10.3389/fmolb.2020.598419)
Supplement: Supplementary file 1 [file datasheet1.pdf]

Supplementary Information

Functional expression of the human glucose transporters GLUT2 and GLUT3 in yeast offers novel screening systems for GLUT-targeting drugs

Sina Schmidl[1], Sebastian A. Tamayo Rojas[1], Cristina V. Iancu[2], Jun-yong Choe[2,3*] and Mislav Oreb[1*]

[1]Institute of Molecular Biosciences, Faculty of Biological Sciences, Goethe University Frankfurt, Frankfurt am Main, Germany

[2]Department of Chemistry, East Carolina Diabetes and Obesity Institute, East Carolina University, Greenville, NC, USA

[3]Department of Biochemistry and Molecular Biology, The Chicago Medical School, Rosalind Franklin University of Medicine and Science, North Chicago, IL, USA

*Corresponding authors

Mislav Oreb
Goethe University Frankfurt
Institute of Molecular Biosciences
Max-von-Laue Straße 9
60438 Frankfurt
Germany
Telephone +49 (0)69 798 29331
Telefax +49 (0)69 798 29527
E-Mail m.oreb@bio.uni-frankfurt.de

Jun-yong Choe
East Carolina University
Department of Chemistry
East Carolina Diabetes and Obesity Institute
105 Heart Drive
ECHI Rm 4114
Greenville, NC27834
USA
Telephone 1-252-744-3368
E-Mail Choej18@ecu.edu

Supplementary Table S1. Strains used in this study

Strain name	Relevant genotype	Reference
EBY.VW4000	*MATa leu2-3,112 ura3-52 trp1-289 his3-1 MAL2-8c SUC2 Δhxt1-17 Δgal2 Δstl1 Δagt1 Δmph2 Δmph3*	Wieczorke et al., 1999
EBY.S7	*MATa leu2-3,112 ura3-52 trp1-289 his3-Δ1 MAL2-8C SUC2 Δhxt1-17 Δgal2 Δagt1 Δstl1 fgy1-1*	Wieczorke et al., 2002
SDY.022	*MATa leu2- 3,112 ura3-52 trp1-289 his3-Δ1 MAL2-8C SUC2 Δhxt1-17 Δgal2 Δagt1 Δstl1 fgy1-1 erg4::kanMX*	Boles et al., 2004
CEN.PK2-1C	*MATa leu2-3,112 ura3-52 trp1-289 his3-Δ1 MAL2–8C SUC2*	Entian and Kötter, 2007

Supplementary Table S2. DNA sequences of the transporters GLUT2 and GLUT3

Shown are wild-type sequences or codon optimized sequences (opt) for expression in insect cells**.**

GLUT transporter	ORF sequence
GLUT2	ATGACAGAAGATAAGGTCACTGGGACCCTGGTTTTCACTGTCATCACTGCTGTGCTGGGTTCCTTCCAGTT<br>TGGATATGACATTGGTGTGATCAATGCACCTCAACAGGTAATAATATCTCACTATAGACATGTTTTGGGTG<br>TTCCACTGGATGACCGAAAAGCTATCAACAACTATGTTATCAACAGTACAGATGAACTGCCCACAATCTCA<br>TACTCAATGAACCCAAAACCAACCCCTTGGGCTGAGGAAGAGACTGTGGCAGCTGCTCAACTAATCACCAT<br>GCTCTGGTCCCTGTCTGTATCCAGCTTTGCAGTTGGTGGAATGACTGCATCATTCTTTGGTGGGTGGCTTG<br>GGGACACACTTGGAAGAATCAAAGCCATGTTAGTAGCAAACATTCTGTCATTAGTTGGAGCTCTCTTGATG<br>GGGTTTTCAAAATTGGGACCATCTCATATACTTATAATTGCTGGAAGAAGCATATCAGGACTATATTGTGG<br>GCTAATTTCAGGCCTGGTTCCTATGTATATCGGTGAAATTGCTCCAACCGCTCTCAGGGGAGCACTTGGCA<br>CTTTTCATCAGCTGGCCATCGTCACGGGCATTCTTATTAGTCAGATTATTGGTCTTGAATTTATCTTGGGC<br>AATTATGATCTGTGGCACATCCTGCTTGGCCTGTCTGGTGTGCGAGCCATCCTTCAGTCTCTGCTACTCTT<br>TTTCTGTCCAGAAAGCCCCAGATACCTTTACATCAAGTTAGATGAGGAAGTCAAAGCAAAACAAAGCTTGA<br>AAAGACTCAGAGGATATGATGATGTCACCAAAGATATTAATGAAATGAGAAAAGAAAGAGAAGAAGCATCG<br>AGTGAGCAGAAAGTCTCTATAATTCAGCTCTTCACCAATTCCAGCTACCGACAGCCTATTCTAGTGGCACT<br>GATGCTGCATGTGGCTCAGCAATTTTCCGGAATCAATGGCATTTTTTACTACTCAACCAGCATTTTTCAGA<br>CGGCTGGTATCAGCAAACCTGTTTATGCAACCATTGGAGTTGGCGCTGTAAACATGGTTTTCACTGCTGTC<br>TCTGTATTCCTTGTGGAGAAGGCAGGGCGACGTTCTCTCTTTCTAATTGGAATGAGTGGGATGTTTGTTTG<br>TGCCATCTTCATGTCAGTGGGACTTGTGCTGCTGAATAAGTTCTCTTGGATGAGTTATGTGAGCATGATAG<br>CCATCTTCCTCTTTGTCAGCTTCTTTGAAATTGGGCCAGGCCCGATCCCCTGGTTCATGGTGGCTGAGTTT<br>TTCAGTCAAGGACCACGTCCTGCTGCTTTAGCAATAGCTGCATTCAGCAATTGGACCTGCAATTTCATTGT<br>AGCTCTGTGTTTCCAGTACATTGCGGACTTCTGTGGACCTTATGTGTTTTTCCTCTTTGCTGGAGTGCTCC<br>TGGCCTTTACCCTGTTCACATTTTTTAAAGTTCCAGAAACCAAAGGAAAGTCTTTTGAGGAAATTGCTGCA<br>GAATTCCAAAAGAAGAGTGGCTCAGCCCACAGGCCAAAAGCTGCTGTAGAAATGAAATTCCTAGGAGCTAC<br>AGAGACTGTGTAA
optGLUT2	ATGACCGAAGACAAAGTGACCGGTACTCTGGTGTTCACCGTGATTACCGCTGTTCTGGGTAGCTTCCAGTT<br>CGGTTATGATATTGGCGTTATCAACGCTCCGCAACAGGTCATCATTAGCCACTATCGCCATGTGCTGGGTG<br>TTCCACTGGATGATCGTAAAGCCATCAACAACTACGTTATCAACAGCACTGATGAACTGCCGACCATTAGC<br>TATAGCATGAACCCAAAGCCGACCCCGTGGGCTGAAGAAGAAACTGTTGCCGCTGCCCAGCTGATTACTAT<br>GCTGTGGAGCCTGAGCGTTAGCAGCTTCGCTGTTGGTGGTATGACCGCCAGCTTCTTCGGTGGTTGGCTGG<br>GTGATACTCTGGGTCGTATCAAAGCCATGCTGGTTGCCAACATCCTGTCTCTGGTTGGTGCTCTGCTGATG<br>GGTTTCTCTAAACTGGGTCCATCTCACATCCTGATCATCGCTGGTCGTTCTATCTCTGGTCTGTACTGTGG

	TCTGATCTCTGGTCTGGTTCCAATGTACATCGGTGAAATCGCTCCAACCGCTCTGCGTGGTGCTCTGGGTA<br>CTTTCCACCAGCTGGCTATTGTTACCGGCATTCTGATCTCTCAGATCATTGGTCTGGAATTCATCCTGGGT<br>AACTACGACCTGTGGCACATCCTGCTGGGTCTGAGCGGTGTTCGTGCTATCCTGCAAAGCCTGCTGCTGTT<br>CTTCTGCCCGGAAAGCCCGCGTTATCTGTATATCAAACTGGATGAAGAAGTCAAAGCTAAACAGAGCCTGA<br>AACGTCTGCGTGGTTACGATGACGTGACCAAAGATATCAACGAAATGCGTAAAGAGCGCGAAGAAGCTAGC<br>AGCGAGCAGAAGGTTTCTATCATTCAACTGTTCACCAACTCTAGCTACCGCCAGCCGATTCTGGTCGCCCT<br>GATGCTGCATGTGGCTCAGCAGTTCAGCGGTATTAACGGTATCTTCTATTATAGCACCTCCATTTTCCAGA<br>CCGCTGGCATCTCTAAACCGGTTTATGCTACCATTGGTGTTGGTGCCGTTAACATGGTGTTCACCGCCGTT<br>AGCGTTTTCCTGGTTGAAAAAGCCGGTCGTCGTTCTCTGTTCCTGATCGGTATGTCTGGTATGTTCGTTTG<br>CGCT<br>ATCTTCATGTCTGTCGGTCTGGTTCTGCTGAACAAATTCTCTTGGATGAGCTACGTTAGCATGATTGCCAT<br>CTTCCTGTTCGTTAGCTTCTTCGAGATCGGTCCGGGTCCGATTCCGTGGTTCATGGTGGCTGAGTTCTTCA<br>GCCAAGGTCCGCGTCCAGCTGCTCTGGCTATTGCTGCTTTCTCCAACTGGACCTGCAACTTCATCGTTGCT<br>CTGTGTTTCCAATACATTGCTGACTTCTGTGGTCCGTACGTGTTCTTCCTGTTCGCTGGTGTTCTGCTGGC<br>TTTCACCCTGTTCACCTTCTTCAAGGTTCCAGAGACCAAAGGCAAGAGCTTCGAAGAAATTGCTGCCGAAT<br>TCCAAAAGAAGAGCGGTTCTGCCCATCGCCCGAAAGCCGCCGTTGAGATGAAATTCCTGGGTGCTACCGAA<br>ACCGTTTAA
GLUT3	ATGGGGACACAGAAGGTCACCCCAGCTCTGATATTTGCCATCACAGTTGCTACAATCGGCTCTTTCCAATT<br>TGGCTACAACACTGGGGTCATCAATGCTCCTGAGAAGATCATAAAGGAATTTATCAATAAAACTTTGACGG<br>ACAAGGGAAATGCCCCACCCTCTGAGGTGCTGCTCACGTCTCTCTGGTCCTTGTCTGTGGCCATATTTTCC<br>GTCGGGGGTATGATCGGCTCCTTTTCCGTCGGACTCTTCGTCAACCGCTTTGGCAGGCGCAATTCAATGCT<br>GATTGTCAACCTGTTGGCTGTCACTGGTGGCTGCTTTATGGGACTGTGTAAAGTAGCTAAGTCGGTTGAAA<br>TGCTGATCCTGGGTCGCTTGGTTATTGGCCTCTTCTGCGGACTCTGCACAGGTTTTGTGCCCATGTACATT<br>GGAGAGATCTCGCCTACTGCCCTGCGGGGTGCCTTTGGCACTCTCAACCAGCTGGGCATCGTTGTTGGAAT<br>TCTGGTGGCCCAGATCTTTGGTCTGGAATTCATCCTTGGGTCTGAAGAGCTATGGCCGCTGCTACTGGGTT<br>TTACCATCCTTCCTGCTATCCTACAAAGTGCAGCCCTTCCATTTTGCCCTGAAAGTCCCAGATTTTTGCTC<br>ATTAACAGAAAAGAAGAGGAGAATGCTAAGCAGATCCTCCAGCGGTTGTGGGGCACCCAGGATGTATCCCA<br>AGACATCCAGGAGATGAAAGATGAGAGTGCAAGGATGTCACAAGAAAAGCAAGTCACCGTGCTAGAGCTCT<br>TTAGAGTGTCCAGCTACCGACAGCCCATCATCATTTCCATTGTGCTCCAGCTCTCTCAGCAGCTCTCTGGG<br>ATCAATGCTGTGTTCTATTACTCAACAGGAATCTTCAAGGATGCAGGTGTTCAAGAGCCCATCTATGCCAC<br>CATCGGCGCGGGTGTGGTTAATACTATCTTCACTGTAGTTTCTCTATTTCTGGTGGAAAGGGCAGGAAGAA<br>GGACTCTGCATATGATAGGCCTTGGAGGGATGGCTTTTTGTTCCACGCTCATGACTGTTTCTTTGTTATTA<br>AAGGATAACTATAATGGGATGAGCTTTGTCTGTATTGGGGCTATCTTGGTCTTTGTAGCCTTCTTTGAAAT<br>TGGACCAGGCCCCATTCCCTGGTTTATTGTGGCCGAACTCTTCAGCCAGGGCCCCCGCCCAGCTGCGATGG<br>CAGTGGCCGGCTGCTCCAACTGGACCTCCAACTTCCTAGTCGGATTGCTCTTCCCCTCCGCTGCTCACTAT<br>TTAGGAGCCTACGTTTTTATTATCTTCACCGGCTTCCTCATTACCTTCTTGGCTTTTACCTTCTTCAAAGT<br>CCCTGAGACCCGTGGCAGGACTTTTGAGGATATCACACGGGCCTTTGAAGGGCAGGCACACGGTGCAGATA<br>GATCTGGAAAGGACGGCGTCATGGAGATGAACAGCATCGAGCCTGCTAAGGAGACCACCACCAATGTCTAA
optGLUT3	ATGGGTACTCAGAAAGTGACTCCAGCTCTGATCTTCGCCATTACCGTTGCCACCATTGGCTCTTTCCAGTT<br>CGGTTACAACACTGGTGTTATCAACGCTCCAGAGAAGATCATCAAAGAGTTCATCAACAAAACCCTGACCG<br>ATAAAGGCAACGCTCCGCCGTCTGAAGTGCTGCTGACCTCTCTGTGGTCTCTGTCCGTTGCCATCTTCTCT<br>GTCGGTGGCATGATTGGCTCCTTCTCTGTGGGTCTGTTCGTTAACCGCTTCGGTCGTCGCAACAGCATGCT<br>GATCGTTAACCTGCTGGCTGTTACTGGTGGTTGCTTCATGGGTCTGTGTAAAGTTGCCAAGAGCGTTGAGA<br>TGCTGATTCTGGGTCGTCTGGTTATCGGTCTGTTCTGTGGTCTGTGCACTGGTTTCGTGCCGATGTATATC<br>GGTGAGATCTCTCCGACTGCTCTGCGTGGTGCCTTCGGCACCCTGAACCAACTGGGTATCGTGGTTGGCAT<br>CCTGGTTGCCCAGATCTTCGGTCTGGAGTTCATTCTGGGTAGCGAGGAACTGTGGCCGCTGCTGCTGGGTT<br>TCACCATTCTGCCAGCTATTCTGCAGTCTGCTGCTCTGCCGTTCTGTCCAGAATCTCCGCGTTTCCTGCTG<br>ATCAACCGTAAAGAGGAGGAGAACGCCAAACAGATCCTGCAACGTCTGTGGGGTACTCAGGACGTTAGCCA<br>GGACATCCAGGAAATGAAAGACGAATCTGCTCGTATGTCTCAGGAGAAGCAGGTTACCGTTCTGGAACTGT<br>TCCGTGTGTCCTCTTACCGTCAGCCGATTATCATCTCCATTGTTCTGCAGCTGAGCCAGCAGCTGTCTGGT<br>ATCAACGCCGTTTTCTACTATTCTACTGGCATCTTCAAAGACGCTGGTGTTCAGGAACCGATTTACGCCAC<br>CATCGGTGCTGGTGTGGTGAACACCATCTTCACCGTGGTTAGCCTGTTCCTGGTTGAACGTGCTGGTCGTC<br>GCACTCTGCACATGATTGGTCTGGGTGGTATGGCCTTCTGCTCTACCCTGATGACCGTTTCTCTGCTGCTG<br>AAAGACAACTACAACGGTATGTCCTTCGTTTGCATCGGTGCCATCCTGGTGTTCGTTGCCTTCTTCGAGAT<br>CGGTCCAGGTCCGATTCCGTGGTTCATCGTTGCTGAACTGTTCAGCCAAGGTCCACGTCCAGCTGCCATGG<br>CTGTGGCTGGCTGTTCCAACTGGACCAGCAACTTCCTGGTGGGTCTGCTGTTCCCGTCTGCTGCTCACTAT<br>CTGGGTGCCTACGTGTTCATCATCTTCACCGGTTTCCTGATCACCTTCCTGGCCTTCACCTTCTTCAAGGT<br>TCCAGAAACCCGTGGTCGTACCTTCGAAGACATCACTCGTGCCTTCGAAGGTCAAGCTCACGGTGCTGATC<br>GCTCTGGCAAAGACGGTGTTATGGAGATGAACAGCATCGAACCAGCCAAAGAAACCACTACCAACGTGTAA

Supplementary Table S3. Primers used in this study

Primer name	Sequence 5'-3'	Application
MOP289	CAAGAACAAACAAGCTCAAC	Sequencing primer forward, binds in *HXT7* promotor region
MOP290	ACCTAGACTTCAGGTTGTC	Sequencing primer reverse, binds in *CYC1* terminator region
MOP425	CAAAAACAAAAAGTTTTTTTAATTTTAATCAAAAAATGACAGAAGATAAGGTCACTG	Amplification of GLUT2 constructs, overhang to *HXT7* promotor, forward
MOP426	ATGTAAGCGTGACATAACTAATTACATGACTCGAGTTACACAGTCTCTGTAGCTCCTAG	Amplification of GLUT2 constructs, overhang to *CYC1* terminator, reverse
MOP427	CACAAAAACAAAAAGTTTTTTTAATTTTAATCAAAAAATGACCGAAGACAAAGTG	Amplification of optGLUT2 constructs, overhang to *HXT7* promotor, forward
MOP428	GAATGTAAGCGTGACATAACTAATTACATGACTCGAGTTAAACGGTTTCGGTAGC	Amplification of optGLUT2 constructs, overhang to *CYC1* terminator, reverse
MOP429	CAAAAACAAAAAGTTTTTTTAATTTTAATCAAAAAATGGGGACACAGAAGGTCAC	Amplification of GLUT3 constructs, overhang to *HXT7* promotor, forward
MOP430	GAATGTAAGCGTGACATAACTAATTACATGACTCGAGTTAGACATTGGTGGTGGTC	Amplification of GLUT3 constructs, overhang to *CYC1* terminator, reverse
MOP431	ACAAAAACAAAAAGTTTTTTTAATTTTAATCAAAAAATGGGTACTCAGAAAGTGACTC	Amplification of optGLUT3 constructs, overhang to *HXT7* promotor, forward
MOP432	GAATGTAAGCGTGACATAACTAATTACATGACTCGAGTTACACGTTGGTAGTGGTTTC	Amplification of optGLUT3 constructs, overhang to *CYC1* terminator, reverse
SSP18	AACTATGTTATCCAAAGTACAGATGAACTGC	Introduce mutation of glycosylation site (N62Q), forward
SSP17	GCAGTTCATCTGTACTTTGGATAACATAGTT	Introduce mutation of glycosylation site (N62Q), reverse
SSP1	CACCATGCTCTGGTCCCTGTCTRTKRYTATKTTTGCAGTTGGTGGAATGACTG	Degenerated primer to introduce a range of mutations in TM2 of GLUT2, forward
SSP2	CAGTCATTCCACCAACTGCAAAMATARYMAYAGACAGGGACCAGAGCATGGTG	Degenerated primer to introduce a range of mutations in TM2 of GLUT2, reverse
SSP3	GATTACTATGCTGTGGAGCCTGAGCRTKRYTATKAGCAGCTTCGCTGTTGGTGGTATGAC	Degenerated primer to introduce a range of mutations in TM2 of optGLUT2, forward
SSP4	GTCATACCACCAACAGCGAAGCTGCTMATARYMAYGCTCAGGCTCCACAGCATAGTAATC	Degenerated primer to introduce a range of mutations in TM2 of optGLUT2, reverse

SSP11	CAGGGACCAGAGCATGGTGATTAGTTGAGCTTTTCGGTCATCCAGTGG	Introduction of Δloop modification to GLUT2, reverse
SSP12	GTTTTGGGTGTTCCACTGGATGACCGAAAAGCTCAACTAATCACCATGCTC	Introduction of Δloop modification to GLUT2, forward
SSP13	CAGGGACCAGAGCATGGTGATTAGTTGAGCAGATCGGTCATCCAGTGG	Introduction of K55S_Δloop modification to GLUT2, reverse
SSP14	GTTTTGGGTGTTCCACTGGATGACCGATCTGCTCAACTAATCACCATGCTC	Introduction of K55S_Δloop modification to GLUT2, forward
SSP15	CAGGGACCAGAGCATGGTGATTAGTTGAGCAGATTTTCGGTCATCCAGTGG	Introduction of ΔloopS modification to GLUT2, reverse
SSP16	GTTTTGGGTGTTCCACTGGATGACCGAAAATCTGCTCAACTAATCACCATGCTC	Introduction of ΔloopS modification to GLUT2, forward
SRP306	ACTACACCTGTAAACAATTCCTCGCCTTTAGACATCACAGTCTCTGTAGCTCCTAG	Amplification of GLUT2 constructs, overhang to *envyGFP*, reverse
SRP218	ATGTCTAAAGGCGAGGAATTG	Amplification of envyGFP, forward
SRP58	TAAGCGTGACATAACTAATTACATGACTCGAG	Amplification of envyGFP, URA3 overhang, reverse

Supplementary Table S4. Plasmids used in this study

Plasmid names	Relevant properties	Reference
p426H7	*2μ, URA3, Amp^r, $HXT7p^{1-392}$, CYC1t*	Mumberg et al., 1995 Hamacher et al., 2002
pRS62K	*2μ, kanMX, Amp^r, $HXT7p^{1-392}$, CYC1t*	Taxis and Knop, 2006; Farwick et al., 2013
pUCPY1	CEN6/ARS4, *URA3, Amp^r, $HXT7p^{1-392}$, CYC1t*	Fernando Garces Daza, Goethe Universität Frankfurt
SSV20	p426H7_Hxt1	This study
SSV16	p426H7_GLUT1	This study
SSV18	p426H7_optGLUT3	This study
SSV19	p426H7_optGLUT3$_{S66Y}$	This study
SSV21	p426H7_GLUT2	This study
SSV23	p426H7_GLUT2$_{\Delta loop}$	This study
SSV26	p426H7_GLUT2$_{K54S_\Delta loop}$	This study
SSV43	p426H7_GLUT2$_{\Delta loopS}$	This study
SSV28	p426H7_GLUT2$_{\Delta loopS_Q455R}$	This study
SSV45	pRS62K_Hxt1	This study
SSV46	pRS62K_GLUT1	This study
SSV47	pRS62K_GLUT2	This study
SSV48	pRS62K_GLUT2$_{\Delta loopS}$	This study
SSV49	pRS62K_GLUT2$_{\Delta loopS_Q455R}$	This study
SSV87	pRS62K_optGLUT3	This study
SSV88	pRS62K_optGLUT3$_{S66Y}$	This study
SSV89	pUCPY1_ GLUT2_envyGFP	This study
SSV90	pUCPY1_ GLUT2$_{\Delta loopS}$ _envyGFP	This study
SSV91	pUCPY1_ GLUT2$_{\Delta loopS_Q455R}$ _envyGFP	This study

Supplementary Table S5. Growth rates and lag phases conferred by different constructs

Apparent maximal growth rates (h^{-1}), calculated with the CGQuant software (Aquila Biolabs) as described previously (Bruder et al., 2016) and lag phases (h), determined from growth curves shown in Figure 1 and Figure 3 are listed. For growth rates, the mean values and standard deviation of triplicates are shown. The duration of lag phases did not differ within one position after decimal point between the replicates. The growth rates are significantly different between GLUT3 and GLUT3$_{S66Y}$ as well as between GLUT2$_{\Delta loopS}$ GLUT2$_{\Delta loopS_Q455R}$ (on both glucose and fructose). The significance was determined by a two-tailed unpaired *t*-test, $p < 0.05$.

Yeast strain	Sugar	Transporter	Max. growth rate (h^{-1})	Lag phase (h)
EBY.S7	0.2 % glucose	Hxt1	0.169 ± 0.036	4.3
		GLUT1	0.117 ± 0.017	7.6
		GLUT3	0.077 ± 0.010	10.7
		GLUT3$_{S66Y}$	0.137 ± 0.002	8.5
		GLUT2$_{\Delta loopS}$	0.049 ± 0.015	13.2
		GLUT2$_{\Delta loopS_Q455R}$	0.084 ± 0.014	12.3
EBY.VW4000	0.2 % glucose	Hxt1	0.214 ± 0.026	3.4
		GLUT3$_{S66Y}$	0.064 ± 0.002	9.9
		GLUT2$_{\Delta loopS_Q455R}$	0.056 ± 0.011	8.5
EBY.S7	2 % fructose	GLUT2$_{\Delta loopS}$	0.14 ± 0.029	12.8
		GLUT2$_{\Delta loopS_Q455R}$	0.21 ± 0.01	17.9

Supplementary Table S6. Effect of phloretin on growth parameters of cells expressing different transporter constructs

Apparent maximal growth rates (h^{-1}), calculated with the CGQuant software (Aquila Biolabs) as described previously (Bruder et al., 2016) and lag phases (h), determined from growth curves shown in Figure 5 (except for Hxt1; not shown) are listed. For growth rates, the mean values and standard deviation of duplicates were calculated. The duration of lag phases did not differ within one position after decimal point between the replicates. Both the growth rates and the lag phases are significantly affected in the presence of phloretin for GLUT2$_{\Delta loopS_Q455R}$. In the case of GLUT3$_{S66Y}$, the growth rates are not significantly different, but the lag phases are. For Hxt1 (control), neither of the growth parameters was significantly affected by phloretin. The significance was determined by a two-tailed unpaired *t*-test, $p < 0.05$.

Yeast strain	Sugar / phloretin		Transporter	Max. growth rate (h^{-1})	Lag phase (h)
EBY.S7	0.2 % glucose	No phloretin	Hxt1	0.169 ± 0.036	4.7
		50 µM phloretin		0.154 ± 0.012	4.7
EBY.S7	0.2 % glucose	No phloretin	GLUT2$_{\Delta loopS_Q455R}$	0.099 ± 0.002	7.1
		50 µM phloretin		0.049 ± 0.008	87.4
EBY.S7	0.2 % glucose	No phloretin	GLUT3$_{S66Y}$	0.128 ± 0.016	6.1
		50 µM phloretin		0.076 ± 0.013	15.6

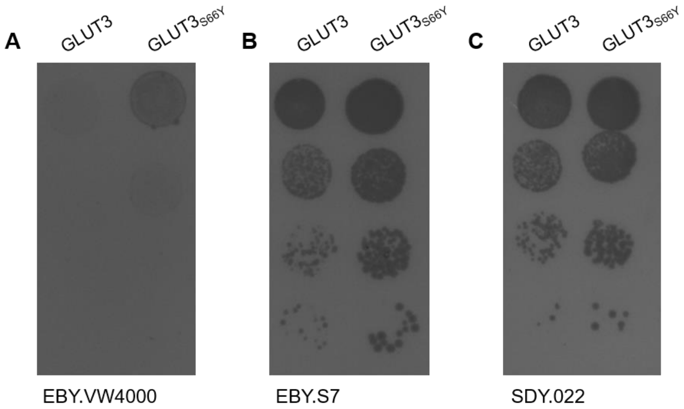

Supplementary Figure S7. Growth test of GLUT3 and GLU3$_{S66Y}$ expressing cells. EBY.VW4000 (A), EBY.S7 (B) or SDY.022 (C) cells were grown over night in SC-URA medium containing 1 % maltose, washed and adjusted to an OD_{600nm} of 1 in ddH_2O. 4 µl of dilutions of OD_{600nm} 1, 0.1, 0.01 and 0.001 were dropped onto SC-URA agar plates with 0.2 % glucose. The plates were incubated at 30°C for 5 days.

```
GLUT1    (64)L W S L S V A I F(72)
GLUT3    (62)L W S L S V A I F(70)
GLUT5    (70)L W S V T V S M F(78)

active       L W S L S V A I F
                 Y V Y M I M
                     T

GLUT2    (96)L W S L S V S S F(104)
```

Supplementary Figure S8. Sequence comparison of the critical TM2 region. Amino acid sequence alignment of the TM2 region, in which mutations required for the functional expression of GLUT1, GLUT3, and GLUT5 in EBY.VW4000 cells frequently occurred. The mutated amino acids (colored) are V69M for GLUT1, S66Y for GLUT3, S72Y or S76I for GLUT5. A summary of the amino acids that occurred in at least one active variant at individual positions is shown in the blue box below the alignment. The most striking difference between GLUT2 and the other GLUT sequences - two consecutive serine residues that align to hydrophobic amino acids in the other three GLUTs - is underlined.

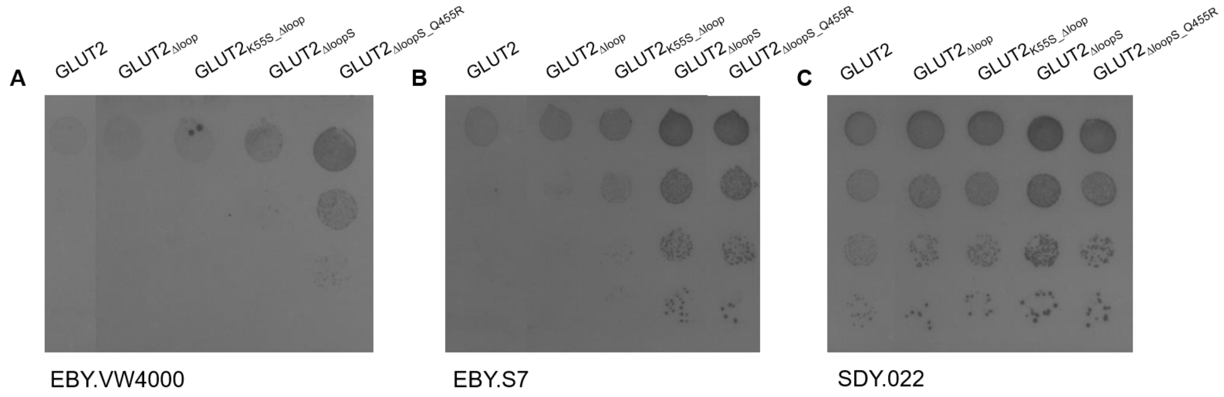

Supplementary Figure S9. Growth test of cells expressing the GLUT2 constructs. EBY.VW4000 (A), EBY.S7 (B) or SDY.022 (C) cells expressing indicated constructs were grown over night in SC-URA medium containing 1 % maltose, washed and adjusted to an OD_{600nm} of 1 in ddH_2O. 4 µl of dilutions of OD_{600nm} 1, 0.1, 0.01 and 0.001 were dropped onto SC-URA agar plates with 0.2 % glucose. The plates were incubated at 30°C for 5 days.

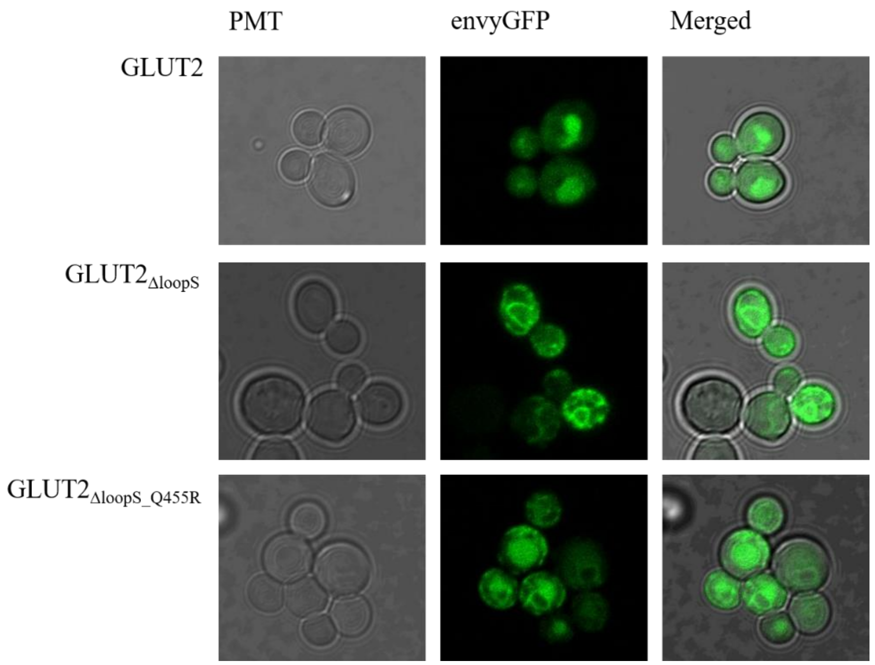

Supplementary Figure S10. Subcellular localization of GLUT2, $GLUT2_{\Delta loopS}$ or $GLUT2_{\Delta loopS\_Q455R}$ in EBY.S7. envyGFP was fused to the C-termini of the three GLUT2 constructs. EBY.S7 cells, expressing the respective envyGFP construct, were grown in low fluorescent SC -URA medium containing 1% maltose. For immobilization 0.6 % low melt agarose was added to the suspension and localization was analyzed with the Confocal Laser Scanning Microscope (Zeiss LSM 780, Jena, Germany).

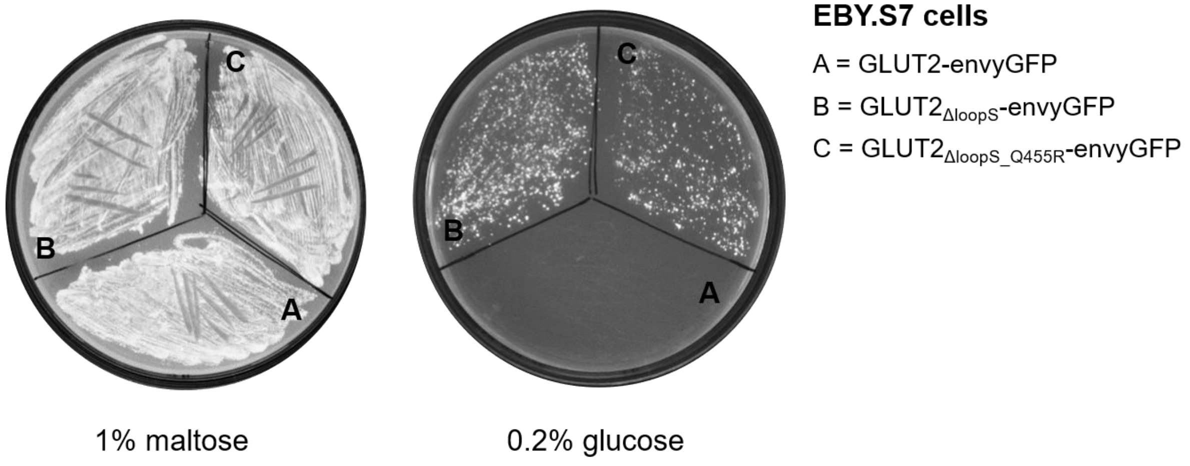

Supplementary Figure S11. Functional expression of the envyGFP-tagged transporter constructs. The C-terminal ends of GLUT2 (A), GLUT2$_{\Delta loopS}$ (B) and GLUT2$_{\Delta loopS_Q455R}$ (C) were fused to envyGFP. EBY.S7 cells expressing the respective envyGFP-tagged transporter construct on a pUCPY1 (CEN.ARS) plasmid were streaked out on solid SC -URA medium containing either 1 % maltose or 0.2 % glucose. The plates were incubated at 30°C for 5 days.

Supplementary References

Boles, E., Dlugai, S., Mueller, G., and Voss, D. (2004). *Use of Saccharomyces cerevisiae ERG4 mutants for the expression of glucose transporters from mammals*. WO002004026907A3. Geneva: World Intellectual Property Organization.

Bruder, S., Reifenrath, M., Thomik, T., Boles, E., and Herzog, K. (2016). Parallelised online biomass monitoring in shake flasks enables efficient strain and carbon source dependent growth characterisation of *Saccharomyces cerevisiae*. *Microb. Cell Fact.* 15, 127. doi: 10.1186/s12934-016-0526-3

Entian, K. D. and Kötter, P. (2007). Yeast Genetic Strain and Plasmid Collections. *Method. Microbiol.* 36, 629–666. doi: 10.1016/S0580-9517(06)36025-4

Farwick, A., Bruder, S., Schadeweg, V., Oreb, M. and Boles, E. (2013). Engineering of yeast hexose transporters to transport D-xylose without inhibition by D-glucose. *Proc. Natl. Acad. Sci. USA* 111, 5159–5164. 10.1073/pnas.1323464111

Hamacher T., Becker J., Gardonyi M., Hahn-Hagerdal B., Boles E. (2002). Characterization of the xylose-transporting properties of yeast hexose transporters and their influence on xylose utilization. *Microbiology* 148, 2783–2788. doi: 10.1099/00221287-148-9-2783

Mumberg, D., Müller, R., Funk, M. (1995). Yeast vectors for the controlled expression of heterologous proteins in different genetic backgrounds. *Gene* 156, 119–122.

Taxis, C., and Knop, M. (2006). System of centromeric, episomal, and integrative vectors based on drug resistance markers for *Saccharomyces cerevisiae*. *BioTechniques* 40, 73–78. doi: 10.2144/000112040

Wieczorke, R., Dlugai, S., Krampe, S., and Boles, E. (2002). Characterisation of mammalian GLUT glucose transporters in a heterologous yeast expression system. *Cell. Physiol. Biochem*. 13, 123–134. doi: 10.1159/000071863

Wieczorke, R., Krampe, S., Weierstall, T., Freidel, K., Hollenberg, C. P., and Boles, E. (1999). Concurrent knock-out of at least 20 transporter genes is required to block uptake of hexoses in *Saccharomyces cerevisiae*. *FEBS Lett.* 464, 123–128. doi: 10.1016/S0014-5793(99)01698-1
